# The Impact of Systemic Inflammation on Alzheimer’s Disease Pathology

**DOI:** 10.3389/fimmu.2021.796867

**Published:** 2022-01-06

**Authors:** Junhua Xie, Lien Van Hoecke, Roosmarijn E. Vandenbroucke

**Affiliations:** ^1^ VIB Center for Inflammation Research, VIB, Ghent, Belgium; ^2^ Department of Biomedical Molecular Biology, Ghent University, Ghent, Belgium

**Keywords:** Alzheimer’s disease, neuroinflammation, peripheral inflammation, systemic inflammation, gut-brain axis

## Abstract

Alzheimer’s disease (AD) is a devastating age-related neurodegenerative disorder with an alarming increasing prevalence. Except for the recently FDA-approved Aducanumab of which the therapeutic effect is not yet conclusively proven, only symptomatic medication that is effective for some AD patients is available. In order to be able to design more rational and effective treatments, our understanding of the mechanisms behind the pathogenesis and progression of AD urgently needs to be improved. Over the last years, it became increasingly clear that peripheral inflammation is one of the detrimental factors that can contribute to the disease. Here, we discuss the current understanding of how systemic and intestinal (referred to as the gut-brain axis) inflammatory processes may affect brain pathology, with a specific focus on AD. Moreover, we give a comprehensive overview of the different preclinical as well as clinical studies that link peripheral Inflammation to AD initiation and progression. Altogether, this review broadens our understanding of the mechanisms behind AD pathology and may help in the rational design of further research aiming to identify novel therapeutic targets.

## Introduction

Dementia is a devastating age-related neurodegenerative disorder that affects over 40 million people worldwide. The speed of disease progression is subjective to individual variability but patients are estimated to live from a few up to 20 years after their diagnosis. Dementia is a very burdensome disorder for patients, their family, caretakers and the health care system as a whole (1). The most prevalent cause of dementia is Alzheimer disease (AD), which is a fatal neurodegenerative disorder that is characterized by progressive cognitive and functional impairment and memory loss. Most AD patients are late-onset and sporadic cases with no proven Mendelian pattern of inheritance. The prevalence of the disease increases with life expectancy and affects 10-30% of people aged over 65 years (2). Recently, the FDA approved Aducanumab which is the first medication that aims at treating AD *via* targeting (one of) the cause(s) of the disease, namely amyloid β (Aβ). However, its therapeutic effect is not yet conclusively proven (3) and next to that only symptomatic medication that is effective for some AD patients is available. Therefore, there is still an urgent need to develop new effective therapies that slow or prevent the progression of AD.

During the past years, the Aβ cascade hypothesis has been the most influential model explaining the pathogenesis of AD. This hypothesis proposes that the extracellular deposition of Aβ in the form of neuritic plaques is the initial pathological event in AD that also leads to the intracellular accumulation of abnormal tau proteins in neurofibrillary tangles (NFTs). These pathological changes, directly or indirectly, induce synaptic and neuronal dysfunction, and ultimately, clinical dementia (4). The steady progress in the understanding of the etiopathogenesis of AD has led to the evaluation of therapies aiming to reduce pathological aggregates of either Aβ or phosphorylated tau (pTau). Unfortunately, none of these strategies has led to clinical success (5–10). As the number of people affected with AD is rising every year, we urgently need to improve our understanding of non-amyloid components and their role in AD pathogenesis. Such new insight may help to identify novel pathways that can be targeted in novel AD therapies.

Over the last years, it became increasingly clear that innate immune activation plays a crucial role in the pathogenesis and progression of AD (11–13). For example, genome-wide association studies (GWASs) show that genes encoding immune receptors such as *CR1*, *CLU*, *CD33*, and *TREM2* are linked with AD development (14). Moreover, a new study also identified *SYK*, *GRN*, *SLC2A5*, *PYDC1*, *HEXB*, and *BLNK* (15), genes involved in the regulation of the immune function within and outside the central nervous system (CNS), as risk genes. Taken together, these studies suggest the role of both the central as well as the peripheral immune system in the development and/or progression of AD.

In this review, we provide a short summary of the different pathways by which the periphery communicates with the brain. Next, we give a comprehensive overview of the different preclinical as well as clinical studies that link peripheral inflammation to initiation and progression of AD. This is followed by an in-depth analysis of the mechanisms of AD pathology that are affected by peripheral inflammation. Lastly, we elaborate on the growing body of evidence indicating that AD may have an underlying intestinal inflammatory process, to which alterations in gut microbiota plays an important role. These insights are essential to broaden our understanding of the mechanisms behind AD pathology and to rationally design new strategies to treat or even prevent the disease.

## How Can the Periphery Communicate With the Brain?

**Figure 1:**
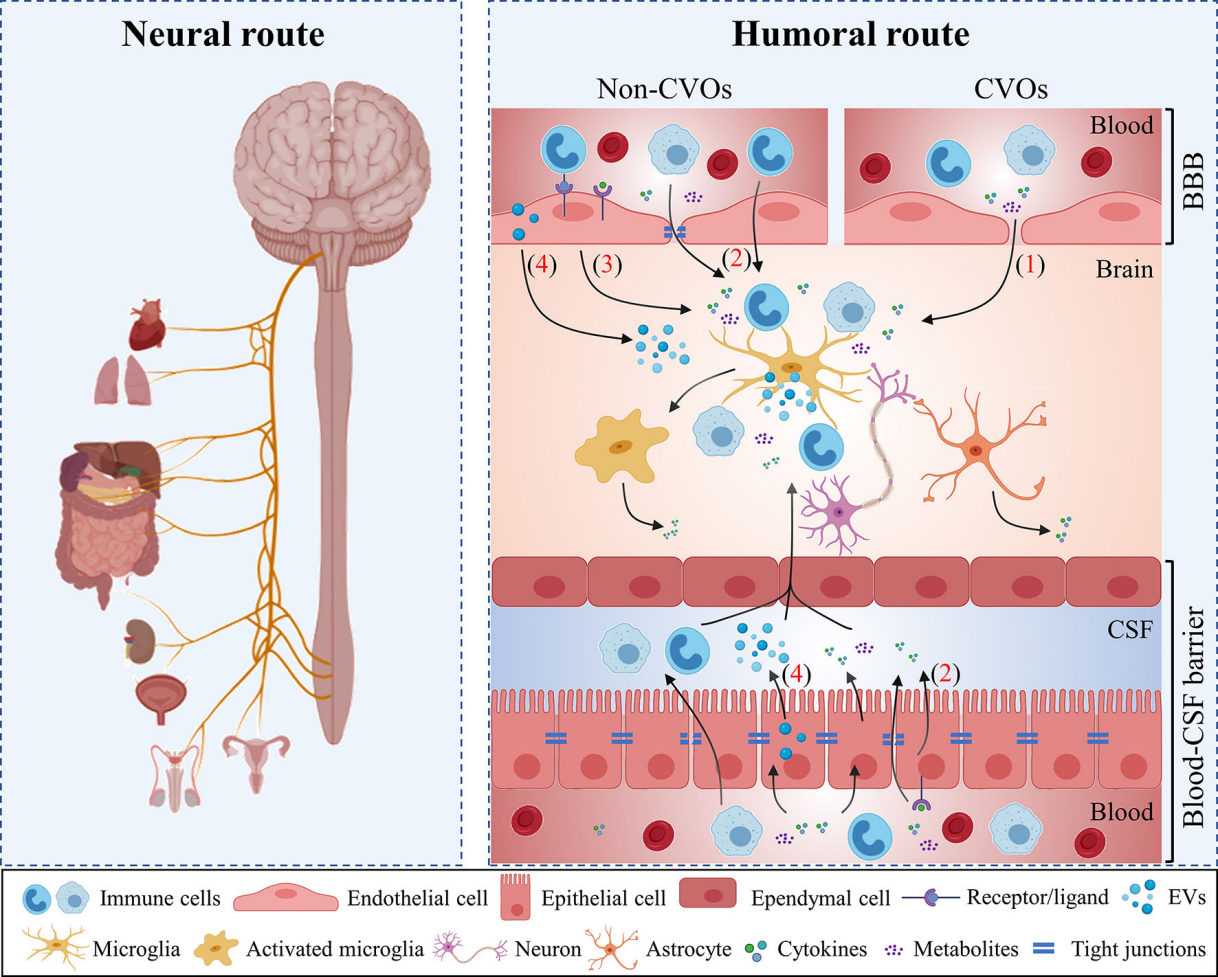
Periphery-to-brain communication pathways. The periphery can communicate to the brain *via* neural and humoral routes. Peripheral organs project signals to varied cerebral regions *via* the vagus nerve (the neural route). Additionally, different humoral routes are used by the periphery to communicate to the brain: (1) Circulating immune mediators access the brain *via* the circumventricular organs (CVOs); (2) Peripheral immune cells cytokines and metabolites interact with their transporters on cerebral endothelial cells and choroid plexus epithelium (CPE) cells and subsequently enter to the brain; (3) Periphery-to-brain communication can occur *via* cell-mediated interactions between peripheral signals and brain cells which in turn lead to microglial activation and neuroinflammation. (4) Peripheral immune mediators activate choroid plexus epithelial cells and induces the release of extracellular vesicles (EVs). EVs enter the brain and can be engulfed by microglia to induce pro-inflammatory response. BBB, blood-brain barrier; CPE, choroid plexus epithelium; CVOs, circumventricular organs; CSF, cerebrospinal fluid; EVs, extracellular vesicles.

The brain is protected from invading substances by tight barriers, including the blood-brain barrier (BBB), the blood- cerebrospinal fluid (CSF) barriers and the arachnoid barrier. These barriers assure a balanced and well-controlled micro-environment in the CNS and they provide protection against external insults such as toxins, infectious agents and peripheral pro-inflammatory cytokines. For this reason, the CNS has long been considered as an ‘immune privileged’ site. However, over the years this idea is been challenged as it is now clear that acute systemic bacterial or viral infections do affect brain functioning (16). Preclinical as well as clinical studies provide evidence that systemic generated inflammatory mediators signal to the brain *via* alternative pathways, namely *via* neural and humoral pathways (**Figure 1**) (17).

### Neural Pathways

Peripheral signals such as cytokines or prostaglandins from the thoracic-abdominal cavity (e.g. Kupffer cells in the liver) directly activate the afferent vagal nerve by binding with the corresponding receptors located at the vagus nerve fiber terminals (16, 18). Subsequently, the vagal nerve signals to the medulla oblongata that on its turn signals to the hypothalamus. This cascade of signaling ultimately leads to changes in neural activity inducing physiological and behavioral responses such as fever and somnolence (16, 18). Important to note is that the efferent vagal nerves can also secrete acetylcholine which on its turn acts on α7 nicotinic receptors expressed on macrophages leading to a downregulation of inflammatory cytokines such as TNF (19). Preclinical evidence shows that vagotomy attenuates the expression of pro-inflammatory cytokines in the brain induced by peripherally administered of LPS or TNF (20). Taken together, these findings underlie the importance of vagal afferents and efferents in the communication between the periphery and the brain.

### Humoral Pathways

Humoral signals, including microbial metabolites, cytokines and immune cells, circulate in the blood and signal to the CNS mainly through the circumventricular organs (CVOs), across the brain barriers or *via* activating vascular cells at the brain barriers (21). *Via* these (in)direct pathways, humoral signals can potentially influence the inflammatory reactions within the brain by modulating the microglial activation, affecting myelination and neurogenesis (22).

CVOs are specialized CNS regions that lack a typical barrier structure as no tight junctions (TJs) between the capillary endothelial cells are present. Consequently, circulating substances from the blood can enter the brain *via* these CVOs (23).

Next to the CVOs, inflammatory molecules and immune cells can also enter the CNS by crossing one of the brain barriers *via* an active transport system (24) or *via* a disrupt barrier (25). Under physiological conditions, only soluble lipid molecules with a low molecular weight (under 0.4-0.6 kDa) and with a positive charge can cross (26). However, during systemic inflammation brain barrier dysfunction may occur and may allow the infiltration of peripheral molecules and immune cells into the CNS. This can then ultimately lead to neuroinflammation (27). Next to the well-known BBB, also the blood-CSF barrier forms an important brain barrier in health and disease. This latter barrier is formed by TJs located between the apical parts of the choroid plexus epithelium (CPE) cells and prevents the accumulation of noxious compounds into the CSF and the brain (28). Recently, it is shown that CPE cells play an important role in the transmission of peripheral signals to the brain (29). In accordance with the BBB, systemic inflammation can disrupt the blood-CSF barrier integrity (29) and leads to the invasion of peripheral immune cells (30). In this regard, Marques et al. showed that the choroid plexus displays an altered expression of genes related to cell entry pathways and innate immune responses upon repeated inflammatory stimuli (31). Interestingly, systemic inflammation also induces the release of pro-inflammatory miRNA-containing extracellular vesicles (EVs) into the CSF. Upon entry into the brain parenchyma, those EVs can be engulfed by *e.g.* microglia and astrocytes and subsequently regulate gene expression thereby transmitting signals from the periphery to the brain (32).

In a third possible humoral route, systemic perivascular macrophages (PVMs) or inflammatory mediators directly activate signaling pathways in vascular cells. This e.g. leads to the release of prostaglandins that are implicated in the development of sickness symptoms such as fever (33).

Of course, the above described pathways are not mutually exclusive, so the net effect of systemic inflammation on the brain can be the result of a combination of the different routes.

## An Increasing Body of Evidence Links Systemic Inflammation to AD Development

Over the last years, it became increasingly clear that systemic inflammation affects the brain in multiple ways and that it is key in the development of neurodegenerative diseases. Below, we look into preclinical and clinical studies that hint to this latter association.

### Evidence From Preclinical Studies

To be able to investigate the effect of systemic inflammatory processes on AD pathology, different mouse models using different insults have been designed. Below we look deeper into these different models and their effect on AD pathogenesis.

The majority of animal models use peripheral injection of immune-stimulating molecules such as lipopolysaccharide (LPS), polyinosinic-polycytidylic acid (poly I:C) or pro-inflammatory cytokines to evoke systemic inflammation. For example, systemic LPS challenge induces a number of sickness symptoms in rodents including fever, loss of appetite and decreased activity (16). Remarkable is that peripheral systemic LPS or increased levels of peripheral pro-inflammatory cytokines do not lead to a prolonged induction of neuronal death (34, 35). It however does lead to temporal induction of neuronal death. This temporary effect can be explained by a whole repertoire of regulatory mechanisms that limit the effects of pro-inflammatory responses in the brain. E.g. increased production of a large variety of proteins from microglial cells including antimicrobial peptides, anti-inflammatory cytokines (IL-10), transforming growth factor (TGF)-β, prostaglandin E2 (PGE2), anti-nuclear factor-kappa B (NF-κB) proteins, mitogen-activated protein and suppressor of cytokine signaling proteins (36). Next to this, the CNS regulates the peripheral inflammatory response through feedback loops in hypothalamic-pituitary-adrenal (HPA) axis and vagal reflex, and neurochemical changes (16). Despite the limited and temporal induction of neuronal damage, a single systemic LPS challenge does lead to increased deposition of Aβ_1-42_ and phospho-tau (p-tau) levels in the brain of wild type rodents (37). Moreover, repeated systemic LPS injections even lead to a prolonged elevation of Aβ levels and cognitive deficits (38–40). Strikingly, pregnant mice exposed to repeated systemic LPS causes AD‐related features including behavioral and neuropathological changes in their offspring (41).

To better understand the impact of systemic inflammation on AD pathology, researcher looked into the effect of systemic inflammation on the removal or the deposition of Aβ plaques in the brain (42). Studies in Tg2576 (43), PDAPP (44), APPSwe (45), APP/PS1 (40, 46) and *App^NL-G-F^* (47) mice show an increase in Aβ deposition upon LPS-induced systemic inflammation. Similarly, poly I:C induced systemic inflammation in 4 months old 3xTg-AD mice increases Aβ deposition in the brain (48). Also, TNF-induced systemic inflammation in 15 months old APP/PS1 mice increases Aβ plaques formation (49). Unfortunately, there are several contradictory results regarding the impact of systemic inflammation on Aβ burden in the mouse brain (**Table 1**). For example, experiments similar to those mentioned above in 3xTg-AD and 5XFAD mice show elevated C-terminal APP fragments (β-CTF) and tau hyperphosphorylation while Aβ deposition is unchanged (59, 60). Even studies in Tg2576 (64, 65, 74), APP/PS1 (50–53) and tgSwe (66) mice show reduced Aβ burden upon systemic LPS challenge. Important to keep in mind is that none of these latter studies analyzed the impact on cognitive impairment. This is important as LPS-induced systemic inflammation affects more than only the Aβ pathology. For example, a study in 3xTg-AD mice demonstrates that LPS challenge affects key neuropathological features of the AD-like phenotype including behavior, microgliosis and astrocytosis (62). In addition, we recently found that low-grade systemic inflammation induced by LPS also causes the microgliosis and neuronal dysfunction in a second-generation mouse model (*App^NL-G-F^*) of AD (47).

**Table 1:** Preclinical studies on the effect of systemic inflammation in mouse models for AD.

Mouse model	Age at first insult	Insult	Stimulus source	Sacrification time after last insult	Effect	Reference
APP/PS1	2 and 12 months	2 µg LPS i.h.	*S. abortus equi*, Sigma	7 days	Decreased Aβ deposits; Increased microglial immunoreactivity; Decreased CD45 immunoreactivity.	(50)
	9 months	0.5 mg/kg LPS i.p. once a week for 13 weeks	*E. coli* 0111:B4, Sigma	4 hours	Decreased Aβ deposits; Increased neuroinflammatory reaction; Elevated lysosomal protease cathepsin Z, APP, APOE, CLU.	(51)
	3 months	3 µg i.p. LPS once per week for 12 weeks	*E. coli* 055:B5, Sigma	Directly after last injection	Increased Aβ deposits.	(46)
	25 months	4 µg LPS i.h.	*S. typhimurium*, Sigma	7 days	Decreased Aβ deposits; Infiltration of BM-derived monocytic cells.	(52)
	11 or 16 months	4 µg LPS i.h.	*S. abortus equi*, Sigma	7 days	Decreased Aβ deposits; Microglial activation.	(53)
	11 ± 4 months	1.5 µg LPS i.p. once per week for 12 weeks	*E. coli* 055:B5, Sigma	Directly after last injection	Increased Aβ deposits; Microglial activation.	(45)
	5 and 15 months	1 mg/kg LPS i.p.	Ultrapure LPS, *S. typhimurium*, #L6143, Sigma.	2 and 10 days	Increased Aβ deposits and impairing microglial Aβ clearance; NLRP3 inflammasome activation.	(40)
	4 and 13–16 months	0.2 mg/kg LPS i.p. once a day for 2 days	*E. coli* O127:B8, #L 3129, Sigma; *S. typhimurium*, #L 6511, Sigma	24 hours and 3 months	Decreased neuronal complexity; Impaired long-term potentiation and spatial learning; NLRP3 inflammasome activation.	(54)
	7 months	5 mg/kg LPS i.p.	*E. coli* 055:B5, #L2880, Sigma	24 hours	Neuronal Apoptosis; Microglial activation; Aggravated cognitive impairment.	(55)
	4.5 months	0.1 mg/kg LPS i.v.	*E. coli* 0111:B4, Sigma	4 hours	Inflammatory reactions; Sex-specific hippocampal metabolic signatures; Sickness behaviors.	(56)
APP23	3 months	10 mg/kg LPS i.p.	*E. coli* 0111:B4, Sigma	0, 1, 3, and 12 hours	Increased neuroinflammatory reaction; Increased vulnerability of the BBB; Severe sickness behaviors.	(57)
	3 months	0.5 mg/kg LPS i.p. once or once a day for 4 days	*S. typhimurium*, Sigma	5 days, 3, 6, and 9 months	Modified pathological features.	(58)
3xtg-AD	3 and 4.5 months	0.25 mg/kg LPS i.p. twice a week for 4 weeks	*E. coli* 0111:B4, Sigma.	Directly after last injection	AβPP β-CTP was increased intraneuronal, but Aβ was unchanged.	(59)
	4 months	0.5 mg/kg LPS i.p. twice per week for 6 weeks	*E. coli* 055:B5, Sigma	24 hours	No effect on Aβ deposits; Inducing tau hyperphosphorylation.	(60)
	4 months	1 mg/kg LPS i.p.	*E. coli* 055:B5, Sigma	6 weeks	long-term impairment on hippocampal neurogenesis and memory.	(61)
	6 months	0.5mg/kg i.p. twice a week for 6 weeks	*E. coli* 055:B5, Sigma	6 weeks	Increased neuroinflammatory reaction; up-regulated γ-secretase; Worsening of behavior; 5-Lipoxygenase pathway affects key neuropathological features.	(62)
	4 months	5 mg/kg Poly I:C i.v.	#P9582, Sigma	11 months	Increased Aβ deposits.	(48)
	5-6 and 11-12 months	Inoculation with 10^4^ tachyzoites of *T. gondii* or 40 viable eggs of *T. muris* by o.g. or i.p. once		5, 7, 9, 35 days	Increased pro-inflammatory response; Increased in immune cell infiltration; Microglial activation.	(63)
Tg2576	17 months	10 µg i.h. LPS	*S. abortus equi*, Sigma	7 days	Decreased Aβ deposits; Microglial activation.	(64)
	16-17 months	4 or 10 µg LPS i.h.	*S. abortus equi*, Sigma	1, 3, 7, 14, 28 days	Decreased Aβ deposits; Microglial and astrocyte activation.	(65)
	6 and 16 months	25 µg LPS i.v.	*E. coli* 0111:B4, Sigma	1, 2, 4, 6, 18 hours	Increased Aβ deposits transiently; Inflammatory responses.	(43)
PDAPP	2 months	10 µg LPS i.c.v. daily for two weeks	*E. coli* 0111:B4, Sigma	Directly after last injection	Increased Aβ deposits; Microglial and astrocyte activation.	(44)
tgSwe	13 months	50 μg LPS i.p.	*E. coli* 055:B5, #L2880, Sigma	1.5 months	Decreased Aβ deposits; Reduced CD45-immunoreactivity.	(66)
5xFAD	3-5 and 13-15 months	0.01, 0.1, 1, 3 mg/kg LPS i.v. once	*E. coli* 0111:B4, Sigma	8 hours	Increased BBB permeability.	(67)
	6 and 13 months	2 µg LPS i.c.v. once or daily	Ultrapure LPS, *P. gingivalis*, *In vivo*Gen	7, 14, 28 days	Increased Iba-1 and CD3 positive cells in periventricular area; No effect on Aβ and cognitive impairment.	(68)
	8 months	Ligature-induced periodontal disease	/	4 weeks	Decreased plaque-associated microglia; Increased insoluble Aβ_42_ level.	(69)
ME7	8 and 19 weeks post-inoculation with ME7	10 μg LPS i.p.	*S. abortus equi*, Sigma	1.5, 3, 6 and 24 hours	Increased levels of IL-1β; Exaggerated sickness behaviors.	(70)
	12, 14 and 15 weeks post-inoculation with ME7	0.1 or 0.5 mg/kg of LPS	*S. abortus equi*, #L5886, Sigma.	2 hours	Exacerbated neuronal death and sickness behavior.	(71)
	18 weeks post-inoculation with ME7	12 mg/kg poly I:C i.p.	Amersham Biosciences	3, 15 hours	Activated interferon-dependent pro-apoptotic pathways; Heightened acute sickness behaviour and acute neurological impairments.	(72)
hAPP-J20	62 weeks	Inoculation with 10^9^ CFU of live *P. gingivalis*	/	5 weeks	Increased Aβ deposits; Trigger neuroinflammation; Enhanced cognitive impairments.	(73)
*App^NL-G-F^*	20-23 weeks	1 mg/kg LPS i.p. once a week for 2 weeks	*S. abortus equi*, #L-5886, Sigma	2 weeks	Increased Aβ deposits; Increased microgliosis; Reduced clearance of Aβ across blood-CSF barrier; Trigger neuronal dysfunction.	(47)

i.h., intrahippocampal; i.p., intraperitoneal; i.c.v., intracerebroventricular; i.v., intravenous.

The contradictory results in literature regarding the effect of systemic inflammation on Aβ pathology can be due to the experimental setup, such as the genetic background of the used mouse model, age, LPS injection procedure such as number of injections, LPS dosage, source and route of administration, and the time between injection and sacrification. **Table 1** gives an overview of preclinical mouse studies in which the effect of systemic inflammation on AD was studied.

Next to the above described genetic mouse models for AD, also ME7 prion mice are used as a model for AD. These mice are characterized by synaptic loss, microglial and astrocyte activation, neuronal death and an age-independent induction of AD pathology (75). A systemic LPS challenge of these ME7 prion mice evokes exaggerated AD related sickness (70). Moreover, a single systemic LPS challenge is enough to cause abnormal CNS functioning including increased pro-inflammatory responses, neuronal apoptosis, cognitive decline, motor symptoms and an earlier onset of AD in the ME7 compared to control mice (35, 71).

Increasing evidence supports the association of systemic inflammation induced by bacterial infections and AD progression in humans. For example, *Helicobacter pylori* (*H*. *pylori*) and periodontal bacteria like *Porphyromonas gingivalis* (*P. gingivalis)* are potential risk factors for the development of AD (16). As the above described immune-stimulating molecules are only mimicking parts of a ‘real’ infection, several studies also use peripheral bacterial infections to induce systemic inflammation.

An experimental study demonstrates that *P*. *gingivalis* infected mice evoke increased expression of IL-1β, AβPP770, cathepsin B (CatB) and Aβ in the liver (76). Additionally, *P*. *gingivalis* infected-hAPP-J20 AD mice, show exacerbated cognitive impairment with increased Aβ deposits and neuroinflammation (73). A variety of *in vivo* studies show that also *H*. *pylori* infection plays a role in AD development. For example, the intraperitoneal injection of *H*. *pylori* TN2GF4 leads to elevated Aβ levels and induces spatial learning and memory impairment in wild type rats (77). The same research group showed that *H*. *pylori* TN2GF4 infection induces significant tau hyperphosphorylation with activation of glycogen synthase kinase-3β (GSK-3β) and that treatment with GSK-3 inhibitors significantly attenuated *H*. *pylori*-induced AD pathology (78). Important to underscore is that these results are obtained upon intraperitoneal injection of *H. pylori* while normally these germs live in the digestive tract. To explore the long-term effect of *H*. *pylori* infection on brain function, *H*. *pylori* TN2GF4 was given this time by oral gavage to rats once a week during a period of four weeks. Despite the reported short-term effects upon intraperitoneal injection, 4 weeks *H*. *pylori* TN2GF4-infected rats *via* oral gavage showed no tauopathy or cognitive impairment. Compared to other *H*. *pylori* strains such as B47 and SS1, the colonization capacity of TN2GF4 is significant lower (79). Consequently, TN2GF4 may not be able to induce a strong inflammatory response resulting in the recovery of the observed short-term pathology (40). However, the route of infection may also play a crucial role on the outcome of the experiments.

Additionally, also the used strain has an impact on the outcome of the infection. For example, infection with *H*. *pylori* B47 or *H*. *felis* CS1 increases neuroinflammation but has no effect on brain Aβ deposition (80) while infection with *H*. *pylori* SS1 or *H*. *felis* has no effect on neuroinflammation nor on Aβ deposition (81). This contradiction can be explained by the fact that different *H. pylori* strains and *H*. *pylori* SS1 lacks some important virulence factors such as functional cag pathogenicity island (cagPAI) and vacuolating cytotoxin A (VacA) (82).

Next to bacterial infections, also viruses and parasites are known to induce systemic inflammation in human and several of them such as herpes viruses and *Toxoplasma* have been proposed as triggers of AD (83, 84). Similarly, also a few mouse studies using viruses or parasites to induce systemic inflammation have been reported in the context of AD. For example, local gut inflammation induced by infection with the parasites *Toxoplasma gondii* and *Trichuris muris* significantly enhances neuroinflammation in 3xTg-AD mice. Unfortunately, no cognitive tests were performed in this study (63). Also *Chlamydophila pneumonia* (*C. pneumoniae*) (85), fungi (86), herpes simplex viruses (HSV) (87) and cytomegalovirus (CMV) (88) are reported to promote the development of AD. Most recently, coronavirus disease 2019 (COVID-19) has been reported to cause various neurologic symptoms including cognitive impairment that may ultimately result in AD, either directly through the invasion of the coronavirus into the CNS or indirectly *via* virus-induced inflammation (89). In rhesus monkey, severe acute respiratory syndrome coronavirus 2 (SARS-CoV-2) was shown to invade primarily *via* the olfactory bulb followed by spreading into CNS thereby inducing neuroinflammation possibly by targeting neurons, microglia, and astrocytes (90).

Surgical interventions are sometimes necessary in human patients for a variety of reasons. In some cases, the immune system may not effectively distinguish between stimuli elicited by surgery and those elicited by trauma or pathogenic infection (91). Surgical procedures thus represent a potential trigger for systemic inflammation and are consequently also used in animal research. A study that uses cecal ligation and puncture (CLP) to induce polymicrobial sepsis in rats shows escalated levels of Aβ, p-tau protein and receptor for advanced glycation end products (RAGE) markers with simultaneous cognitive impairment (92). Similarly, laparotomy-induced systemic inflammation in wild-type mice induced abnormal tau phosphorylation and memory impairment (93). Furthermore, orthopedic surgery causes hippocampal-dependent memory impairment, which is associated with increased levels of IL-1β both in plasma and hippocampus (94).

Also for several non-infectious diseases such as atherosclerosis, obesity, diabetes and depressive illness, there is clear evidence for being risk factors in the development of AD and all these diseases are associated with a chronic pro-inflammatory phenotype (16). For example, in B6Tg2576 mice, a transgenic mouse model of AD was produced by back-crossing Tg2576 mice, early atherosclerosis lesions were detected and were positively correlated with brain Aβ accumulation when mice were fed a normal diet or atherogenic diets (95, 96). Diabetic mouse models include streptozotocin-induced Type 1 diabetes and high-fat and/or sugar diet-induced Type 2 diabetes have been showed to aggravate Aβ pathology in APP transgenic mice and even in non-transgenic rodents (97). Similarly, high fat diet-induced obesity in mice promotes systemic inflammation and impairs cognitive functioning through increased Aβ accumulation and BBB disruption (98, 99). For the studies on depression in mouse models of AD, anti-depressive treatment can decrease Aβ burden and cognitive impairment (100).

Altogether, the above described preclinical studies form a large body of evidence showing an association between peripheral inflammation and AD pathology. Rodent studies suggest that Aβ and neuroinflammation are the result of a direct response to systemic infections and are part of the brains innate immune response to inflammatory insults. Of note, every mouse model has limitations in replicating the full AD pathology, and further side-by-side comparisons between different mouse models and observations from human AD patients are required to move towards the development of effective treatments.

### Evidence From Clinical Studies

Next to the preclinical studies, there are also a large number of clinical studies reporting that systemic inflammation is associated with the increase in cognitive decline in AD (101–103). This systemic inflammation can be caused by specific environmental factors, bacterial and viral infections and the presence of a chronic disease, such as diabetes.

#### Environmental Risk Factors

It is well known that several of the environmental risk factors for the development of AD have systemic pro-inflammation as a common characteristic. For example, ageing is a major risk factor and is accompanied by a low-grade systemic inflammation and a relative decline in adaptive immunity and T helper 2 (Th2) cell response. This concept is better known as inflammaging (16) and is caused by an imbalance between pro- and anti-inflammatory mediators. An example of inflammaging is the age-related endocrine dyscrasia with loss of sex steroids and elevation of gonadotrophins that is associated with an increased pro-inflammatory state and is remarkably also associated with an increased development of AD (104, 105).

There is also a build-up of amyloid plaques throughout life. However, it is not clear if this build-up is primarily caused by the occurrence of systemic inflammatory events or if it is due to other ageing mechanisms or even a combination of both. Cross-sectional studies show that patients with cognitive impairment and evidence of Aβ exhibit an increased systemic inflammatory response and increased microglial activation compared to healthy subjects (106). Nevertheless, it is also known that not all these patients have activated microglia (107) and that one-third of the healthy population older than 80 years have Aβ loads comparable to the load found in AD patients (108). Next to this, persons with high Aβ loads but without dementia have lower level of pro-inflammatory cytokines than AD patients (16). Additionally, analysis of AD and mild cognitive impairment (MCI) patients showed a significant correlation between cognition and microglial activation but not with Aβ loads (109, 110). These findings support the idea that microglial activation rather than Aβ alone may be the key change leading to initiation and progression of AD.

#### Bacterial and Viral Infections

Along with preclinical studies, also epidemiologic studies demonstrate an association between periodontal bacteria or *H*. *pylori* infection and MCI and AD (111–113). GWAS show that *P*. *gingivalis* infection significantly enrich the expression of genes related to cognitive decline (114). Moreover, AD patients show elevated levels of antibodies against periodontal bacteria (115, 116), increased levels of periodontopathic virulence factors (117) and *P*. *gingivalis* in postmortem brain tissue (118). Also, gingipains, i.e. proteases secreted by *P. gingivalis*, are found in the brain of 90% of AD patients and this is correlated with the present tau and Aβ levels (118). Taken together, all these data suggest that periodontitis leads to cognitive decline that may be mediated by systemic inflammation (119). In contrast, a recent cross-sectional study reports that only 11 of the 29 inflammatory biomarkers are associated with cognitive impairment in patients with severe periodontitis. However, the inflammatory response to severe periodontitis was more reduced (lower biomarker concentrations) in patients with cognitive impairment or dementia than in cognitively healthy controls (120). These contradictory or inconsistent results may be caused by differences in study design, diagnostic criteria, length of follow-up, controls, and appropriate analytical approach (121).

Next to periodontal bacteria, also *H*. *pylori* is a possible risk factor for the development of AD, although not all studies are consistent about this (122). Clinical studies confirm that AD and MCI patients have higher anti-*H*. *pylori* IgG titers in their blood and brain (111, 123) and indicate that AD patients have more gastric inflammation (112). Moreover, the presence of *H*. *pylori* IgG antibodies is associated with a 1.46 times increased risk for the development of dementia compared to non-infected controls (124). Additionally, two independent surveys show that *H*. *pylori* eradication may improve AD manifestation at 2- and 5-year clinical endpoints (125, 126). In contrast to the above studies is the study of Roubaud-Baudron et al. that shows that there is no correlation between *H*. *pylori* infection and the occurrence of AD (127). Important to keep in mind is that the latter study has a small patient cohort and lack non-AD dementia patients or control patients. Moreover, serum antibody detection was used to diagnose *H. pylori* infection but this method has a high rate of false negative result (128).

Next to the effect of bacterial infections, increasing clinical evidence indicates that also viral and fungal infections may be causative factors for the development of AD. For example, neurotropic pathogens such as HSV-1 have been repeatedly isolated from the brains of AD patients and HSV infection is associated with a higher risk for dementia in some studies (129–132). In contrast to these studies, a genetic analysis study using Mendelian randomization found no association between gene-predicted risk for herpes infection and subsequent cognitive decline or AD (133). The same holds true for the association between an infection with cytomegalovirus (CMV) and the development of dementia (134).

#### Chronic Diseases

The chronic diseases including obesity, diabetes, Atherosclerosis and depressive illness have also an epidemiological basis for being proposed as risk factors in the development of AD and are all associated with a chronic pro-inflammatory state. The abnormal metabolism inherent to a chronic disease induces a general pro-inflammatory response in peripheral organs. For example, dysfunction of lipid metabolism in obese patients leads to inappropriate overflow of circulating free fatty acids (FFAs), which can activate pro-inflammatory pathways through cell surface pattern recognition receptors (PRRs) (135). Consequently, this sustained chronic inflammatory situation can amongst others disrupt blood-brain interfaces, allowing peripheral mediators to enter the brain. This on its term might induce persistent chronic brain inflammation resulting in problems with brain function, including cognitive impairment in AD (22). In obese and diabetic patients, they show a greater cognitive decline over last decades compared to healthy subjects (136–139). The further investigation indicates the higher blood glucose levels in the preceding 5 years correlate with an increased risk of dementia among participants with and without diabetes (140). Epidemiological studies also report that the incidence cardiovascular disease affects Aβ metabolism and leads to accumulation of Aβ in peripheral and brain (141). Depression is common in AD patients, but the individual with depression is also associated with higher chance in dementia risk (100). While their individual attributable risk is likely to be small, their combined cumulative effects over time might be considerable (16).

## Systemic Inflammation Affects Different Characteristics of AD Pathology

**Figure 2:**
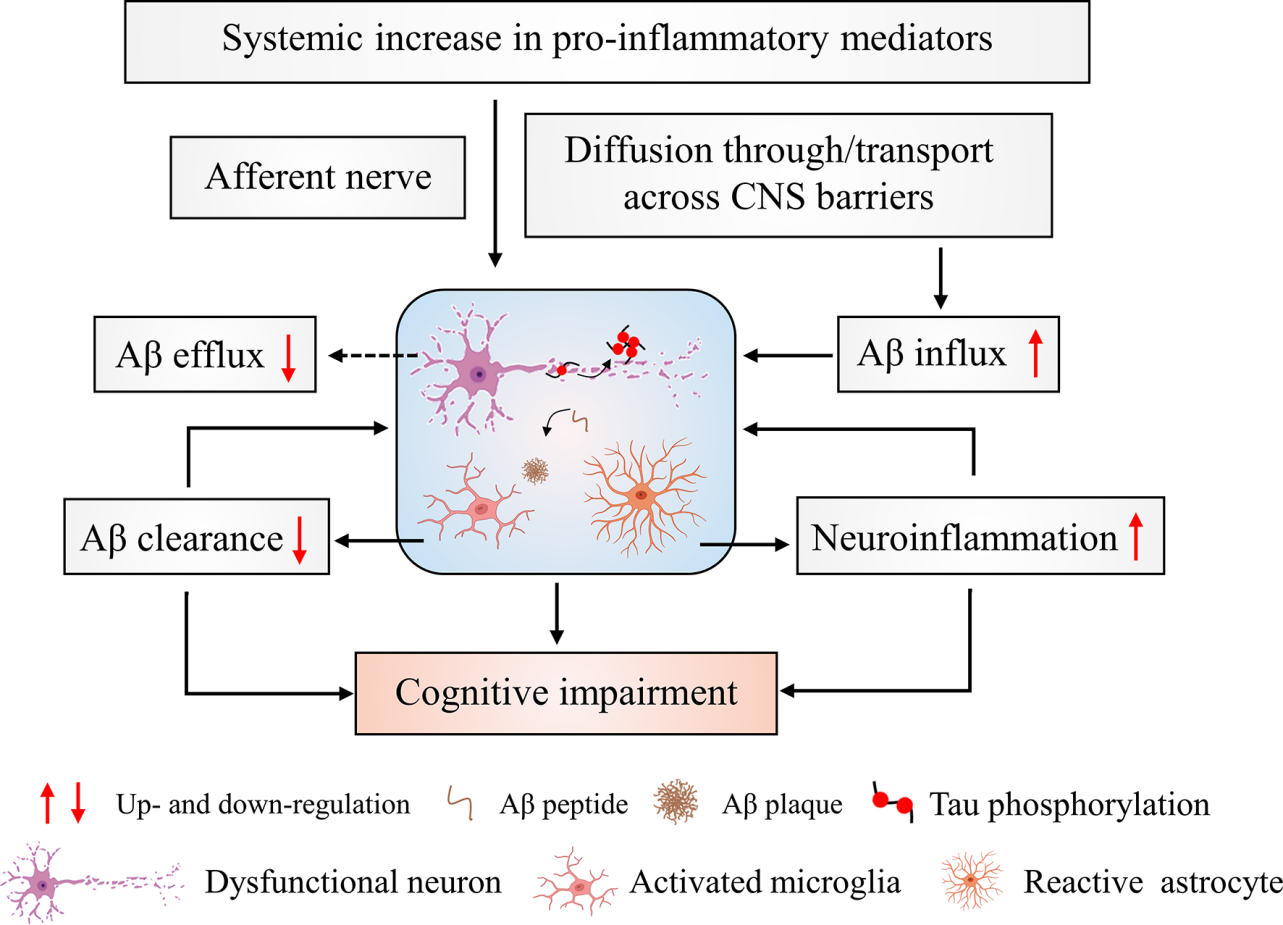
Cerebral changes in response to systemic inflammation. Systemic inflammation leads to increased levels of pro-inflammatory mediators. These signals can project to the brain *via* nerve afferents and the brain barriers. This can directly and/or indirectly induce neuronal cytotoxicity and affect Aβ transport resulting in increased Aβ aggregation. The aggregated Aβ induces an initial activation of microglial cells that leads to activated microglial cells with an impaired Aβ clearing ability. Additionally, the activated microglial cells produce a large amount of pro-inflammatory cytokines that further exacerbate neuroinflammation. This worsening of neuroinflammation promotes the development of brain pathology and ultimately leads to cognitive impairments.

As discussed above there is a huge amount of (pre)clinical data supporting the hypothesis that systemic inflammation is correlated with AD pathology. Below, we look deeper into the mechanisms of AD pathology that are affected by systemic inflammation. More precisely, we elaborate on how systemic inflammation induces neuroinflammation, promotes Tau hyperphosphorylation, impairs Aβ clearance and brain barrier integrity (**Figure 2**). The mechanism studies included below are mainly from preclinical experiments, but were applicable also clinical studies are mentioned.

### Systemic Inflammation Triggers Neuroinflammation

Peripheral inflammation results in the activation of the innate immune system and the production of pro-inflammatory cytokines. These immune factors circulate in the blood and ultimately affect neurons and glial cells in the brain *via* the neural and humoral pathways mentioned above. In the context of chronic inflammation present in AD patients, evidence suggests that peripheral immune cells infiltrate in the CNS and accumulate near areas of pathology (142). On their term, these cells induce increased microglial activation and Aβ deposition (143). Microglia, the primary immune effector cells of the CNS, are key cellular mediators of the neuroinflammatory responses in AD (144). In absence of inflammatory stimuli, microglial cells are in a “resting” or inactive state in which they continually survey their immediate environment without interfering with neurons and neuronal activities. However, when activated, the morphological and biological functions of microglial cells are altered and they migrate to the site of injury to initiate an innate immune response (145, 146). Unfortunately, when microglia remain for a long time in such an activated state, they release cytokines and neurotoxic agents such as IL-1β, TNF, IL-6, nitric oxide (NO) and reactive oxygen species (ROS) that can directly or indirectly cause neuronal cell death (144).

Microglia express various cell surface receptors, such as PRRs that recognize misfolded and aggregated proteins such as Aβ and subsequently trigger an innate immune response (147). Activated microglia that are persistent in the release of pro-inflammatory mediators are involved in the suppression of axonal transport and adult neurogenesis (148). Moreover, activated microglia are characterized by the retraction of their processes, which is a phenotypic change that correlates with an impaired ability to remodel synapses (47, 149). This effect contributes to impaired synaptic plasticity seen in AD. Additionally, prolonged microglia impairment caused by pro-inflammatory cytokines can also damage neurons by reducing trophic factors such as brain-derived neurotrophic factor (BDNF) and insulin-like growth factor (IGF) (149–151). Studies on signaling mechanisms indicate that microglial myeloid differentiation primary response 88 (MyD88) (152) and p38 mitogen activated protein kinase (MAPK) (153, 154) signaling are involved in the release of neurotoxins leading to neuronal damage. These inflammatory effects are specifically initiated by the microglia-derived pro-inflammatory cytokine TNF (153, 155). In addition, neuronal mitochondria play an important role in the regulation of microglial activation and the neuronal protein Mitofusin-2 is likely the mechanistic linker between neuronal mitochondria dysfunction and neuroinflammation (156).

Next to microglia also inflammasomes, i.e. multiprotein complexes that control the production of pro-inflammatory cytokines, play an important role in neuroinflammation (157). The NOD-, LRR- and pyrin domain-containing 3 (NLRP3) inflammasome is one of the best defined and most widely implicated regulators of IL-1β and IL-18 production (157). Inflammasomes function as intracellular sensors for both foreign and host-derived danger signals. For example, soluble Aβ oligomers and protofibrils can induce NLRP3 inflammasome activation in microglia (158). Ablation of NLRP3 inflammasomes protects mice from age-related increases of innate immune activation in the periphery and the CNS and attenuates functional decline (159).

In addition, also other molecular and cellular players are involved in the institution of neuroinflammation upon peripheral inflammation. For example, a study demonstrates that IL-32 and IL-32β affect neuroinflammation and Aβ formation by activating signal transducers and activators of transcription 3 (STAT3) and NF-κB (160).

### Systemic Inflammation Impairs Aβ Clearance

Aβ load is the net result of Aβ production and Aβ clearance and small changes in this equilibrium result in abnormal accumulation of the protein. Aβ clearance depends on several potential pathways (1): phagocytosis (2), endocytosis and macropinocytosis by professional phagocytes and microglia, as well as by astrocytes, oligodendrocytes and neurons (3), Aβ degradation by Aβ-degrading enzymes like neprilysin, insulin-degrading enzyme (IDE) and matrix metalloproteinases (MMPs) and (4) Aβ efflux from the brain to the blood and influx from the blood to the brain *via* transport across the BBB and blood-CSF barrier, interstitial fluid bulk flow and CSF egress pathways, including arachnoid villi and glymphatic-lymphatic pathways (47, 161).

In aged AD mice, microglial cells have lower expression of Aβ-phagocytic receptors and Aβ-degrading enzymes, but their ability to produce pro-inflammatory cytokines is maintained (162). Furthermore, AD mice crossed with Tg197 mice (163), mice carrying a modified human TNF-globin transgene, show deregulated patterns of human TNF gene expression that develop chronic inflammatory polyarthritis and amyloid deposition. The increased amyloid deposition can be explained by the fact that systemic TNF indirectly modulates Aβ pathology by regulating peripheral immune cell trafficking and glial responses in the brain (164). Our recent study has also indicated that sustained exposure to LPS leads to impaired microglial phagocytosis of Aβ and increased Aβ deposition in *App^NL-G-F^* mice (47).

Recently, it has been shown that inflammasome signaling is involved in microglial Aβ clearance. Indeed, NLRP3 deficient APP/PS1 mice are partially protected from Aβ pathology and neuronal dysfunction under LPS-induced systemic inflammation (40, 54). Importantly, these mice show almost normal cognitive function and they are protected from Aβ-induced suppression of synaptic plasticity (165). ASC (apoptosis-associated speck-like protein containing a C-terminal caspase recruitment domain) is a key adaptor molecule required for the inflammatory processes and represents an essential step in the activation of NLRP3 inflammasomes (166). Aβ can bind to ASC and consequently amplifies NLRP3 inflammasome activity which on his turn impairs Aβ clearance by microglia. The resulting accumulated Aβ binds to more ASC and in this way a vicious circle is established (167).

Next, low Aβ levels within the healthy brain are also maintained through transport across the BBB (161). Unfortunately, LPS-induced systemic inflammation causes lipoprotein receptor-related protein-1 (LRP-1)-dependent decreased Aβ entry into the blood (168, 169) and increased Aβ influx into the brain (170). Moreover, polymicrobial infection-induced RAGE accumulation facilitates the transport of Aβ across the BBB and increases the central Aβ load (92). In addition, the blood-CSF barrier is also involved in processes that clear substances from the CSF and the blood. Moreover, the main transporters of Aβ are also found in choroid plexus epithelium, including LRP1, LRP2, P-glycoprotein (P-gp) and RAGE (47, 171). Interestingly, using primary cells to mimic the blood-CSF barrier, we recently showed that the transporter LRP2 is involved in Aβ efflux from the CSF to blood side and that this is impaired in response to inflammation (47).

### Systemic Inflammation Induces Changes at the Brain Barriers

As already mentioned above, the brain parenchyma is enclosed by different structures including multilayered meninges, the BBB, the blood-CSF barrier and the glia limitans. The BBB and blood-CSF barrier are the two main brain barriers to impede free diffusion between brain and blood and to provide transport processes for essential nutrients, ions and metabolic waste products (172). Although research is mainly focused on the BBB, more and more research is now emphasizing the important role of the blood-CSF barrier in CNS homeostasis and neurological disorders. The BBB is formed by endothelial cells that are tightly linked by tight junctions (TJs). On the basement side of the membrane, pericytes and astrocytes perform supporting and regulatory functions (173). The blood-CSF barrier is formed by TJs between neighboring choroid plexus epithelial cells (174).

As described above, a number of *in vitro* and *in vivo* studies show that systemic inflammation has disruptive effects on BBB integrity leading to the diffusion of peripheral inflammatory factors into the brain (25). These factors then further induce changes in the brain and are associated with an increased cognitive decline in AD patients (16). Next to the diffusion of peripheral factors, also Tau transmission in the brain *via* the disrupted BBB is observed upon peripheral inflammation (175).

A number of mechanisms are described to explain the BBB disruptive effect of systemic inflammation. Central to these mechanisms are prostanoids and NO, which are both synthesized by LPS-stimulated cerebrovascular endothelium and surrounding cells (25). The mediators, including MMPs (176, 177) and ROS (178), are also involved in the destruction of BBB integrity by activating intracellular pathways such as MAPK signaling (176) and inducing mitochondrial dysfunction (179) while blood-CSF barrier integrity loss was linked to MMP-dependent collagen cleavage (173). In addition, some miRNAs play a role in BBB structure and function (180). E.g. the expression of miR-155 in microvessels is strongly and rapidly upregulated by inflammatory cytokines and alters BBB function by affecting expression of TJs and adhesion components (181).

Next to these molecules, also different cell types influence BBB integrity. Abbott and colleagues showed that astrocytes and pericytes secrete a number of molecules that enhance and maintain BBB integrity. Moreover, the end-feet of these cells form a lacework of fine lamellae closely linked to the outer surface of the BBB endothelium and basement membrane (182). Previous studies have shown that systemic inflammation induces astrocyte proliferation and activation followed by astrocyte loss (183, 184) and changes in astrocytic end-feet structures (185). During sustained systemic inflammation, astrocytes produce a range of substances including pro-inflammatory cytokines and prostaglandins that are associated with disruption of the BBB (25). Next, also microglia are important players of the neurovascular unit although their ablation in mature mice does not directly leads to an increase in BBB permeability (150). However, a recent study shows that vessel-associated microglia initially maintain BBB integrity *via* expression of claudin-5 and make physical contact with endothelial cells. Yet, under LPS-induced systemic inflammation, brain resident microglia migrate in a CCR5-dependent manner to the cerebral vasculature and phagocytose astrocytic end-feet. In this way microglia then anyway impair BBB integrity (186).

The crossing of leukocytes through the BBB upon systemic inflammation primarily occurs at postcapillary venules and may occur in a paracellular or transcellular way (25). TNF has been shown to promote expression of brain microvessel P- and E-selectin, which are both required for cellular recruitment (187). In addition, systemic inflammation stimulates endothelial production of chemokines such as CCL2, which lead to conformational changes in leukocyte integrins and enhance their binding to endothelial ligands (188). Similarly, the expression of endothelial cell adhesion molecules such as intercellular adhesion molecule 1 (ICAM-1) are promoted upon inflammation. Important is that leukocytes need to cross the glia limitans to enter the brain parenchyma after passing though the endothelium and this crossing depends on the degradation of basement membrane components by MMP-2 and -9 (189). *In vitro* studies show that LPS stimulation increases expression of MMP-2 in the endothelium (176) and pericytes (190).

Next to direct morphological changes in the BBB, systemic inflammation can also cause non-disruptive changes that affect BBB functionality. For example, there are different studies indicating that BBB transport pathways are affected by systemic inflammation. Including the downregulation of the multi-functional efflux transporter P-gp (191) and the upregulation of influx carriers responsible for TNF (192). Correspondingly, systemic inflammation also results in a reduced bulk flow of CSF and interstitial fluid (ISF) across the BBB, which can further impair Aβ clearance (168). Additionally, non-disruptive BBB changes during systemic inflammation may also promote neuroinvasion of pathogens. For example, systemic LPS enhances the transcellular transport of the human immunodeficiency virus (HIV) without disrupting the BBB though luminal stimulation by IL-6 and granulocyte-macrophage colony-stimulating factor (GM-CSF) and MAPK signaling (193).

As mentioned above, systemic inflammation not only has an impact on the integrity of the BBB, but LPS-induced systemic inflammation in mice also disrupts the blood-CSF barrier (47, 177) and induces the release of extracellular vesicles (EVs) (32); both resulting in increased neuroinflammation. Interestingly, also Aβ on its own has a direct impact on the blood-CSF barrier: induction of morphological changes of the CPE cells, decreased expression of TJ components, loss of barrier integrity, and EV release into the CSF (47, 194, 195). Analysis of human gene expression data comparing control and AD patient choroid plexus tissue revealed that TNF/TNFR1 signaling was upregulated in AD suggesting an involvement of this pathway in the blood-CSF barrier associated changes in AD (196). In agreement with this, both TNFR1 deficiency and treatment with a TNFR1 inhibitor prevented the Aβ-induced cognitive decline (196). Additionally, also treatment with an inhibitor of EV production had the same effect (195). Based on these results, it is tempting to speculate that the combined effect of systemic inflammation and the presence of Aβ might further worsen AD disease progression among others *via* specific mechanisms at the blood-CSF barriers, such as loss of barrier integrity and EV release, which on their turn further aggravate neuroinflammation. However, in our study in which *APP^NL-G-F^* mice were challenged twice with a low dose of LPS no additive effect on blood-CSF barrier disruption was observed (47), which might indicate that high LPS levels are needed to disrupt this barrier.

### Gut Inflammation Affects AD Pathology

Despite the anatomical separation between the CNS and the gastrointestinal system, a bidirectional network between the two, known as the ‘gut-brain axis’, exists. A growing body of evidence indicates that AD may have an underlying intestinal inflammatory process, to which altered gut microbiota plays an important role. The gut-brain interaction can occur *via* two routes: *via* vagal transmission and *via* systemic circulation (197).

Thousands of sensor and motor fibers from the vagus nerve connect the gut with the brainstem and serve as a conduit for neural signals. These signals are governed by changes in enteric neuron activity and the behavior of gut microbes. Such gut bacteria activate the vagus nerve either directly or indirectly through their metabolites and neuroactive substances. Moreover, the vagus nerve interacts extensively with different components of the systemic immune system and in this way continuously monitors the inflammatory state of the gut. Upon sensing inflammatory signals by binding to specific receptors in the vagus nerve fiber such as the proinflammatory tachykinin/neurokinin receptors (198), the vagal afferents transmit signals to the dorsal vagal complex (DVC) where most sensory information is relayed to nucleus tractus solitarii (NTS) (199). Neurons in the NTS that receive the vagal sensory inputs can modulate microglial state and activity by specific ligand-receptor pairs (e.g. CX3CL1-CX3CR1 and CD200-CD200R), neurotransmitters (e.g. glutamate and GABA), and purinergic signaling (200). Activated microglia on their turn produce pro-inflammatory (e.g. nitric oxide and PGE2) and cytokines (e.g. TNF, IL-1β and IL-6) and ultimately influence the neuroinflammatory state (201). Concurrently, vagal efferent nerves hand over information about the immune status of the brain back to the gut, with increased CNS inflammation feeding back to inhibit further release of peripheral pro-inflammatory cytokines through acetylcholine-mediated signaling (202). Effective vagal nerve signaling is critical for sending appropriate signals to microglia in order to modulate levels of neuroinflammation (201). Moreover, vagus nerve stimulation combined with LPS challenge leads to a decrease in microglial production of pro-inflammatory cytokines in the brain, an effect no longer observed after vagotomy (203). Taken together, the vagus nerve is a physical conduit between gut microbial activity and neuroinflammation.

Interestingly, intestinal inflammation induced by gut microbiota perturbation is directly associated with intestinal barrier dysfunction. The enhancement of intestinal permeability allows the entrance of pathogenic, immune-stimulating and neuroactive substances into the systemic circulation. Increased systemic pro-inflammatory cytokines and neurotoxic compounds may contribute to an increased microglial activation and production of pro-inflammatory cytokines in the brain as described above (204, 205). Calprotectin as a marker of intestinal inflammation and has intrinsically amyloidogenic amino acid sequences that can form amyloid oligomers and fibrils (206). Interestingly, calprotectin levels are significantly increased in the CSF and the brain of AD patients, which promotes its amyloid aggregation and co-aggregation with Aβ (207). It is possible that this intestinal source of calprotectin may contribute to amyloid fibril formation in the gut or directly in the brain.

Along with affecting the level of intestinal permeability, gut microbiota can indirectly influence the state of systemic inflammation through interactions with nearby immune cells. When pathogen-associated molecular patterns (PAMPs) produced by pathogenic invaders bind to PRRs, such as TLRs, inflammatory cytokine production is altered (208). The circulation and subsequent potential entry of these cytokines into the brain act locally on CNS cells, including microglia, thereby influencing the state of inflammation in the brain (209). Indeed, increased intestinal inflammation driven by either LPS or bacterial infection correlates with elevated levels of microglial activation and release of pro-inflammatory cytokines (124, 210).

Gut microbiota-derived metabolites are additional contributors to the gut-brain crosstalk. Circulation of microbial metabolites, including neurotransmitters, short-chain fatty acids (SCFAs), and trimethylamines can potentially influence microglial activation through direct and indirect means (197, 211). For example, binding of serotonin to 5-hydroxytryptamine receptors expressed on microglia induces the release of cytokine-carrying exosomes and induces neuroinflammation (212). However, the exact SCFA signaling pathways that modulate microglial activation are not yet fully understood. Mice lacking the free fatty acid receptor 2 (FFAR2), a GPCR required for SCFA signaling in the gut, exhibit a microglial phenotype similar to that observed in germ-free mice (209). The absence of FFAR2 expression on microglia suggests that SCFAs may influence microglial activation through signaling that originate in the gut and SCFAs may have potential direct influences on microglia. Indeed, treatment of microglial cells *in vitro* with various SCFAs, including valproic acid and butyric acid, elevates the levels of acetylation of histone H3 (213). This suggests that SCFAs influence microglial behavior *in vivo* through a combination of GPCR signaling and histone deacetylase inhibition.

Bacterial extracellular vesicles (bEVs), including Gram-negative bacteria derived outer membrane vesicles (OMVs) and Gram-positive bacteria secreted membrane vesicles (MVs), carry molecular cargo from paternal bacteria to target cells. bEVs contain numerous PAMPs, including DNA, RNA, lipoproteins, LPS and peptidoglycan. The PAMP content of bEVs enables them to engage with host PRRs and consequently initiate pro-inflammatory signaling cascades that lead to the production of cytokines and chemokines. A growing body of evidence suggests that bEVs play a key role in the communication and regulation of the host and even manipulate the host immune response. Interestingly, this may ultimately affect AD progression (214, 215). For example, OMVs from *H*. *pylori* were shown to modulate pro-inflammatory responses in gut epithelial cells with a dose-dependent production of the pro-inflammatory cytokine IL-8 (216). Also bEVs from other bacteria have immunostimulatory abilities in mice and human (214). Taken together, bEVs have the abilities to modulate systemic inflammation and disrupt epithelial barrier integrity and ultimately increase development of AD though the pathways mentioned above. For example, extracellular RNAs (exRNAs) in periodontal bacteria *Aggregatibacter actinomycetemcomitans* OMVs were shown to cross the BBB in mice and promote TNF production in human macrophages *via* activating TLR-8 and NF-κB signaling pathways (217). In addition, intravenous administration of bEVs isolated from faeces of AD patients increases the BBB permeability and promotse glial cell activation in wild-type mice, thereby inducing an inflammatory response and tau hyperphosphorylation by activating the GSK‐3β pathway and finally leading to cognitive impairment (218).

All the above mentioned findings further underscore the associated between systemic inflammation, influenced in part by the gut microbiota, and BBB, microglial activation and neuroinflammation, and ultimately AD.

## Conclusion

Increasing evidence indicates that systemic inflammation might drive the initiation and progression of AD. It is becoming increasingly clear that the brain cannot longer be viewed as an immune-privileged region and that CNS inflammation and systemic inflammation are connected to each other. Systemic inflammation rather than Aβ or tau alone may be a key player in AD pathology and its role may precede Aβ deposition. In addition, whether the build-up of Aβ plaques and tau hyperphosphorylation as we age is primarily due to the occurrence of systemic inflammatory events or to other ageing mechanisms is still unclear but it is likely that the combination of different factors, such as inflammaging and microglial priming are crucial. Clearly, continued research in this area is needed to further unravel the effects of systemic inflammation on AD and its mechanisms. Furthermore, this review strengthens the believe that peripheral inflammation worsens AD progression and this opens up a wide range of possible therapeutic strategies for AD *via* the modulation of peripheral inflammation.

## Author Contributions

JX wrote the manuscript. LVH and RV critically revised the manuscript. All authors have read and agreed to the published version of the manuscript.

## Funding

JX is supported by the Chinese Scholarship Council (CSC) grant number: 201808360194. Research in the authors’ lab is funded by the Foundation for Alzheimer’s Research Belgium (SAO-FRA) grant number: 20200032, the Baillet Latour Fund and the Research Foundation Flanders (FWO Vlaanderen) G055121N.

## Conflict of Interest

The authors declare that the research was conducted in the absence of any commercial or financial relationships that could be construed as a potential conflict of interest.

## Publisher’s Note

All claims expressed in this article are solely those of the authors and do not necessarily represent those of their affiliated organizations, or those of the publisher, the editors and the reviewers. Any product that may be evaluated in this article, or claim that may be made by its manufacturer, is not guaranteed or endorsed by the publisher.
